# Correlates of the “No-Reflow” or “Slow-Flow” Phenomenon in Patients Undergoing Primary Percutaneous Coronary Intervention 

**Published:** 2018-07

**Authors:** Mohammad Alidoosti, Reza Lotfi, Masoumeh Lotfi-Tokaldany, Ebrahim Nematipour, Mojtaba Salarifar, Hamidreza Poorhosseini, Arash Jalali

**Affiliations:** *Tehran Heart Center, Tehran University of Medical Sciences, Tehran, Iran.*

**Keywords:** *Myocardial infarction*, *Percutaneous coronary intervention*, *No-reflow phenomenon*

## Abstract

**Background: **Despite recent advances in interventional equipment and techniques, the angiographic no-reflow phenomenon occurs in a considerable number of patients undergoing primary percutaneous coronary intervention (PCI). We investigated the clinical, angiographic, preprocedural, and procedural characteristics associated with the no-reflow phenomenon among patients undergoing primary PCI.

**Methods:** Between March 2008 and April 2013, 530 patients (78.5% male, mean age=58.11±12.39 y) with ST-segment-elevation myocardial-infarction who underwent primary PCI were categorized in 2 groups according to their postprocedural thrombolysis-in-myocardial infarction (TIMI) flow grades: those with a maximum score of 2 (the no-reflow or slow-flow group) and the ones with a score of 3 (the reflow group). A multivariable logistic regression model was used to find the multiple correlates of the no-reflow phenomenon after PCI.

**Results:** There were 166 (31.3%) patients in the no-reflow group and 364 (68.7%) in the reflow group. The no-reflow patients were older and had significantly longer target lesion lengths, higher SYNergy between percutaneous coronary intervention with TAXus and cardiac surgery (SYNTAX) scores, higher infarct-related artery SYNTAX scores, more thrombus burden, and a higher frequency of initial TIMI flow grades of 2 or lower. Our multivariable logistic regression analysis demonstrated that older age, higher numbers of Q waves, not using statin, longer target lesion lengths, higher thrombus grades, and higher infarct-related artery SYNTAX scores remained the independent correlates of increased no-reflow rates after primary PCI (area under the ROC curve=0.784, 95% CI: 0.742–0.826; P<0.001).

**Conclusion:** Clinical, angiographic, and procedural features of patients undergoing primary PCI may be correlated with the occurrence of the no-reflow phenomenon. The thrombus grade and the infarct-related artery SYNTAX score could be among these factors.

## Introduction

Early and adequate revascularization of an infarct-related artery (IRA) plays a crucial role in the management of acute ST-elevation myocardial infarction (STEMI), and primary percutaneous coronary intervention (PCI) is an established mainstay in the urgent reperfusion of an IRA in STEMI.^1^^, ^^2^ However, successful revascularization, demonstrated by angiography, does not necessarily maintain adequate reperfusion in the myocardium.^3^^, ^^4^ Defined by angiography, the no-reflow phenomenon is manifested by an acute reduction in the coronary flow (thrombolysis in myocardial infarction [TIMI] flow grade=0–1) in the absence of dissection, thrombus, spasm, or high-grade residual stenosis at the original lesion site. Lesser degrees of flow impairment (TIMI score=2) are generally referred to as “slow flow”.^2^ TIMI flow grades of 2 or lower are associated with an increased incidence of death and myocardial infarction.^5^ For all the recent advances in interventional equipment and techniques, the angiographic no-reflow phenomenon occurs in a considerable number of patients undergoing primary PCI, ranging between 12% and 32.8%.^6^^-^^9^ The predisposing factors for the no-reflow phenomenon are not fully understood. Several clinical, angiographic, and echocardiographic measures have been proposed as factors associated with the development of the no-reflow phenomenon in different studies with differing designs.^10^^-^^12^ The most recent studies have introduced newer preprocedural angiographic associate factors such as the SYNergy between percutaneous coronary intervention with TAXus and cardiac surgery (SYNTAX( score and thrombus burden in separate settings; however, the independent effect of each factor still needs further investigation.^6^^, ^^8^^, ^^9^

In the present study, we aimed to investigate the clinical and preprocedural characteristics associated with the no-reflow phenomenon among patients undergoing PCI on native coronary arteries for STEMI. 

## Methods

The present retrospective observational study was conducted in Tehran Heart Center, Tehran Iran. Between March 2008 and April 2013, a total of 607 primary PCI procedures were performed. Seventy-seven patients were excluded because they had stenting on grafted arteries. The remaining 530 patients were included in this study. The diagnosis of STEMI was confirmed by coronary angiography in all the patients, and primary PCI was carried out within 24 hours of symptom onset. Patients with an unsuccessful primary PCI and pretreatment with fibrinolysis before primary PCI were excluded. In addition, because of the apparent effect in the coagulation/ fibrinolysis system, patients with severe liver or renal disease, neoplasm, or hematological disorders were eliminated from the study. Also excluded from the study were patients who received glycoprotein IIb/IIIa inhibitors (because during the time period of patient selection, only a few patients received IIb/IIIa inhibitors when no the reflow phenomenon occurred after primary PCI). Previous coronary artery bypass graft surgery was considered the exclusion criterion because firstly the SYNTAX score (which is one of our proposed angiographic features) could not be calculated in these patients and secondly saphenous vein grafts are more prone to the no-reflow phenomenon after primary PCI.^13^^, ^^14^ The protocol of the current study was approved by our institutional review board, and it was performed in compliance with the guidelines on human studies and those by the Food and Drug Administration (USA). All the patients provided a written consent form at the time of admission, allowing the researchers to use their medical information for research purposes. 

The diagnosis of STEMI was based on chest pain lasting for at least 30 minutes and elevated ST segment more than 1 mm in at least 2 consecutive electrocardiographic (ECG) leads confirmed by a further increase in the level of serum creatine kinase and/or troponin. Pre-infarction angina was defined as a typical angina chest pain within a 48-hour period prior to infarction. Detailed data on the patients’ medical history and physical examination, as well as their procedural and postprocedural information, were prospectively entered in the hospital’s PCI Data Registry. The definitions of the variables in the registry can be found in our previous reports.^15^^, ^^16^

All the patients underwent urgent diagnostic coronary angiography according to the standard criteria. Significant coronary artery disease was defined as the presence of at least 70% luminal diameter stenosis in at least 1 epicardial coronary artery.

PCI was performed using a standard femoral approach with a 6- or 7-F guiding catheter. All the patients received 300 mg of oral aspirin and 300 mg of clopidogrel immediately after admission and 5000 U of intravenous heparin before PCI. All the patients were successfully revascularized on the IRA after coronary angiography. Aspiration thrombectomy was performed in more than 2 passages across the lesion. The intra-aortic balloon bump (IABP) was inserted in most patients with cardiogenic shock or some patients in Killip class 3 that needed IABP support before PCI.

All the angiograms were evaluated by 2 experienced interventional cardiologists, who did not have knowledge of the clinical and laboratory data. Coronary thrombus burden was scored in 5 grades based on angiographic evaluation. The classifications of the TIMI thrombus grades, according to a previous definition,^17^ are shown in Table 1. Thrombus burden was classified as mild if the TIMI thrombus was class 0 and 1, moderate if the TIMI thrombus was class 2 and 3, and high if the TIMI thrombus was above class 3.^18^ The patients were divided into 2 groups according to their postprocedural TIMI flow grades: those with a maximum score of 2 (the no-reflow or slow-flow group) and the ones with a score of 3 (the reflow group).

The infarct-related artery SYNTAX score (IRA-SS) was defined as IRA lesions with greater than 50% diameter stenosis in a vessel larger than 1.5 mm (all the treated vessels in this study had a diameter >1.5 mm), based on the SYNTAX Score Calculator 2.1 (available at http://www.syntaxscore.com). 

**Table 1:** Thrombolysis-in- myocardial infarction (TIMI) grading of thrombus burden

TIMI grade 0: no angiographic evidence of thrombus
TIMI grade 1 (possible thrombus present): reduced contrast density, irregular lesion contour, or a smooth convex meniscus at the site of the total occlusion, suggestive but not diagnostic of thrombus
TIMI grade 2 (small thrombus): definite thrombus, with greatest dimension <1/2 the vessel diameter
TIMI grade 3 (moderate thrombus): definite thrombus but with greatest linear dimension >1/2 but <2 the vessel diameter
TIMI grade 4 (large thrombus): definite thrombus, with the largest linear dimension >2 the vessel diameter
TIMI thrombus grade 5: total occlusion

Adapted with permission from Sianos G, Papafaklis MI, Serruys PW. Angiographic thrombus burden classification in patients with ST-segment elevation myocardial infarction treated with percutaneous coronary intervention. J Invasive Cardiol 2010;22(Suppl B):6B-14B.

The continuous variables were presented as means and standard deviations or medians with interquartile ranges and were compared between the reflow and no-reflow groups using the Student *t* or Mann–Whitney test. The categorical variables were reported as frequencies and percentages and were compared between the 2 mentioned groups using the χ^2 ^test or the Fisher exact test. A multivariable logistic regression model with the backward method (probability of 0.05 for entry and 0.1 for removal) was used to find the multiple correlates of the no-reflow phenomenon after PCI. Variables with a p value less than 0.2 in the univariate analyses were candidated for the multivariable model. The effect of the variables on the no-reflow phenomenon was reported through odds ratios with 95% confidence intervals. The discrimination power of the model was measured using the area under the receiver operating characteristics (ROC) curve. The calibration of the model was tested using the Hosmer–Lemeshow test. All the statistical analyses were done using IBM SPSS Statistics for Windows, version 20.0 (Armonk, NY: IBM Corp.).

## Results

A total of 530 patients, comprising 416 men and 114 women at a mean age of 58.11±12.39 years, were included for analysis. The mean total SYNTAX score was 24.54±9.86, and the mean target vessel SYNTAX score was 15.94±8.62. The study population was divided into 2 groups according to their postprocedural TIMI flow grades. There were 166 (31.3%) patients in the no-reflow group and 364 (68.7%) patients in reflow group. The baseline and presenting characteristics of both groups are depicted in Table 2. According to Table 2, the patients in the no-reflow group were older, more frequently presented with anterior wall STEMI, and had more Q waves in the surface ECG. The use of preprocedural statins in the no-reflow group was significantly less than that in the reflow group. There were no significant differences in the coronary artery disease risk factors and history of previous myocardial infarction. 

With regard to the angiographic and procedural data presented in Table 3, the left anterior descending artery (LAD) accounted for the majority of the IRAs in both groups; however, this was more frequently observed in the no-reflow group than in the reflow patients (65.1% vs. 50.3%, respectively). The no-reflow patients had significantly longer target lesion lengths, higher IRA SYNTAX scores, higher frequencies of high-grade thrombus burden, and higher initial TIMI flow grades of at least 2. The patients in the no-reflow group were less frequently subjected to stent implantation (82.5% vs. 93.7% in the reflow group; P<0.001). Drug-eluting stents were used in 36.1% of the patients with no reflow and in 40.9% with reflow; the difference constituted no statistical significance. The implantation of multiple stents was found more frequently in the no-reflow group (P=0.052).

Multivariate logistic regression analysis became possible with the inclusion of the following variables: age, hypertension, history of previous myocardial infarction, anterior STEMI, number of Q waves, use of statins prior to primary PCI, IRAs, lesions in the proximal portion of the LAD, target lesion lengths, initial TIMI flow grades, thrombus grades, multiple stent implantation, and (IRA-SS). The results of the multivariate analysis showed that older age, higher numbers of Q waves in the surface ECG, not using statin, longer target lesion lengths, higher thrombus grades, and higher IRA-SS were the independent factors significantly associated with the increased rate of the no-reflow or slow-flow phenomenon after primary PCI (for the model, area under the ROC curve=0.784, 95% CI: 0.742–0.827; P<0.001) (Table 4). 

**Table 2:** Baseline and presenting characteristics of the study patients in the reflow and no-reflow groups*

	No-Reflow Group (n=166)	Reflow Group (n=364)	P
Age (y)	59.89±12.32	57.30±12.34	0.025
Men	136 (81.9)	280 (76.9)	0.193
Diabetes mellitus	48 (28.9)	104 (28.6)	0.935
Hypertension	86 (51.8)	220 (60.4)	0.062
Hyperlipidemia	95 (57.9)	219 (60.5)	0.578
Smoking cigarettes	55/160 (34.4)	132/361 (36.6)	0.631
Family history of CAD	23 (13.9)	66/363 (18.2)	0.227
Body mass index, kg/m^2^	27.00±3.47	26.97±3.97	0.927
Previous myocardial infarction	5 (3.0)	23 (6.3)	0.114
Pre-infarction angina	144 (87.3)	320 (87.9)	0.836
Pre-infarction angina class III/IV	133 (80.6)	295 (81.0)	0.906
Anterior STEMI	80 (48.2)	138 (37.9)	0.026
Number of ECG leads with the Q wave	3 (0.4)	2 (0.3)	<0.001
Use of statins	150 (90.4)	347 (95.3)	0.028

CAD, Coronary artery disease; STEMI, ST-segment elevation myocardial infarction; ECG, Electrocardiography

* Data are presented as mean±SD or n (%).

**Table 3:** Angiographic and procedural data in the reflow and no-reflow groups*

	No-Reflow Group (n=166)	Reflow Group (n=364)	P
Number of diseased vessels			0.359
One-vessel disease	48 (28.9)	112 (30.8)	
Two-vessel disease	67 (40.4)	124 (34.1)	
Three-vessel disease	51 (30.7)	128 (35.2)	
Infarct-related coronary artery			0.006
LAD	108 (65.1)	183 (50.3)	
LCX	20 (12.0)	59 (16.2)	
RCA	38 (22.9)	122 (33.5)	
Target lesion location			0.307
Ostial	17 (10.2)	27 (7.4)	
Proximal	84 (50.6)	172 (47.3)	
Mid or distal	65 (39.2)	165 (45.3)	
Lesion in the proximal part of the LAD	64 (38.3)	103 (28.3)	0.018
AHA grade B2/C	143 (86.7)	303 (83.2)	0.316
Target lesion length (mm)	24.88±11.21	21.97±9.65	0.004
Reference vessel diameter (mm)	3.18±0.48	3.16±0.49	0.681
Initial TIMI flow ≤2	150 (90.4)	256 (70.3)	<0.001
Thrombus burden			<0.001
Low	20 (12.0)	110 (30.5)	
Moderate	55 (33.1)	182 (50.0)	
High	91 (54.8)	71 (19.5)	
Use of drug-eluting stents	60 (36.1)	149 (40.9)	0.338
Use of bare-metal stents	77 (46.4)	192 (52.7)	0.174
Stenting after pre-dilation	105(63.3)	253(69.5)	0.154
Post-dilation balloon	81 (48.8)	203 (55.8)	0.135
Additional balloon dilation	6 (3.6)	7 (1.9)	0.241
Multiple stent implantation	21 (12.7)	27 (7.4)	0.052
Infarct-related SYNTAX score	19.40±8.69	14.36±8.13	<0.001

AHA, American Heart Association; LAD, Left anterior descending artery; LCX, Left circumflex artery; RCA, Right coronary artery; TIMI, Thrombolysis in myocardial infarction

* Data are presented as mean±SD or n (%).

**Table 4:** Independent associated factors of the no-reflow phenomenon after primary PCI

Variable	Odds ratio	95% confidence interval	P
Age (for a 1-year increase)	1.032	1.014-1.051	0.001
Hypertension	0.653	0.422-1.011	0.056
Number of Q waves (for 1 increase)	1.122	1.019-1.236	0.020
History of previous MI	0.381	0.130-1.122	0.080
Use of statins	0.411	0.18-0.938	0.035
Lesion length (for 1 mm increase)	1.025	1.004-1.046	0.017
Thrombus grade (for a 1-grade increase)	1.881	1.570-2.253	<0.001
The infarct-related artery SYNTAX score	1.042	1.016-1.069	0.002

P value for the Hosmer–Lemeshow test=0.935, area under the receiver operating characteristics curve=0.784 with 95% confidence interval=0.742–0.826 and P<0.001

## Discussion

In the present study, the rate of the no-reflow phenomenon after primary PCI for STEMI was 31.3%. We found that older age, higher numbers of Q waves in the surface ECG, not using statin, longer target lesion lengths, higher thrombus grades, and higher IRA-SS were independently correlated with the increased probability of the no-reflow phenomenon after primary PCI.

The cause of the no-reflow phenomenon after primary PCI in patients with STEMI is complex. The possible mechanisms of this phenomenon include endothelial dysfunction, microvascular dysfunction, spasm, embolization, and reperfusion injury.^19^^, ^^20^ Different sample sizes and inclusion criteria for the selection of study populations have resulted in dissimilar no-reflow among various studies.^6^^-^^8^^, ^^10^^, ^^21^ We found an incidence rate of 31.3% for myocardial no-reflow, which is consistent with the reported rate of 32.8% by Sahin et al.^9^ Elsewhere, the incidence of myocardial no-reflow among patients with anterior STEMI, detected by myocardial contrast echocardiography, was 39.6%,^10^ while other investigators reported incidence rates of 12%, 14.3%, and 24.3% in patients undergoing primary PCI within 12 hours of symptom onset.^6^^, ^^7^^, ^^18^ The no-reflow phenomenon, defined as TIMI flow grades of 0 or 1, was found in 2.5% of patients with STEMI in a study by Harrison et al.^21^ In another study, the reported incidence for female patients was 25.3%, where angiographic no-reflow was defined as a TIMI flow grade below 3 or 3 with a blush grade of 0 to 1.^8^

It has been documented that the pre-revascularization angiographic features of the IRA such as scored thrombus burden can be drawn upon as a simple and efficient clinical tool in the prediction of the slow-flow or no-reflow phenomenon after PCI.^22^^, ^^23^ Although some of the previous investigators have not included the effect of thrombus burden in their study,^6^^, ^^10^ the reports by other studies in different settings indicate that a higher preprocedural thrombus grade of the IRA affects the flow restoration and increases the risk of the no-reflow phenomenon.^7^^, ^^8^^, ^^17^^, ^^24^ In the present study, a higher preprocedural thrombus grade was one of the independent correlates for the no-flow or slow-flow phenomenon after primary PCI for acute STEMI.

The SYNTAX score is an angiographic grading tool to determine the complexity of coronary artery disease, and it has been shown to be able to aid revascularization and predict mortality and morbidity at both short- and long-term follow-ups.^25^ The usefulness of the SYNTAX score to identify patients at risk of the no-reflow phenomenon after primary PCI has been reported by a small number of studies.^6^^, ^^9^ The overall SYNTAX score indicates the global burden of atherosclerosis in a given myocardium (even the burden of non-IRAs); accordingly in this study, we included only IRA-SS and observed that the no-reflow group had a significantly higher IRA-SS at the time of the initial diagnostic angiography and this factor remained an independent factor significantly associated with no-reflow occurrence in our study. 

A study by Sahin et al.^9^ showed that the involvement of the LAD was an independent correlate of myocardial no-reflow. Margo et al.^6^ stated that infarction in the proximal LAD was allied with a 3.5-fold risk of angiographic no-reflow. This association was also reported between the culprit lesion in the proximal part of the LAD and echocardiographic myocardial no-reflow by Iwakura et al.^10^ We observed that the patients with the involvement of the LAD had a higher rate of developing the no-reflow phenomenon than those with the involvement of the left circumflex artery or the right coronary artery in the univariable analysis. However, our multivariate analysis failed to show the involvement of the LAD as an independent correlate for the no-reflow phenomenon. 

The extent of the ischemic region and myocardial damage is considered an important risk indicator for myocardial no-reflow. Kloner et al.^26^ demonstrated that the no-reflow region of the myocardium was located within the necrotic area, where severe myocardial damage had caused some kind of no-reflow prior to reperfusion. According to the findings by Iwakura et al.,^10^ the number of Q waves in the surface ECG (as an indicator of the severity and extent of myocardial damage) and the wall motion score index (indicator of the size of the risk area) were independently correlated with the development of the no-reflow phenomenon detected by myocardial contrast echocardiography. These observations were confirmed by our results in as much as the angiographic myocardial no-reflow was independently correlated with a higher number of Q waves in the preprocedural ECG in our patients. 

A meta-analysis of 7 studies with 3086 patients revealed that acute intensive statin therapy before PCI significantly reduced the hazard of postprocedural no-reflow.^11^ Although more than 93% of our study patients received statin therapy prior to primary PCI, the frequency of the patients receiving statin was significantly lower in those with the no-reflow phenomenon (90% vs. 95%, respectively) and the independent relationship between statin use and the no-reflow phenomenon was also significant in our study. 

The independent relationship between age and no-reflow development, which was found in the present study, has also been reported by previous investigators.^6^^, ^^9^^, ^^18^ In-hospital and long-term mortality rates are higher in elderly patients with acute myocardial infarction, and the success rate of primary PCI is lower in these patients than in younger patients because of delayed hospitalization.^27^^-^^29^ Diffuse coronary atherosclerosis, severe vascular calcification, distal embolization, and microcirculation dysfunction are more common in elderly patients. These pathological changes are related to advanced age, absence of ischemic preconditioning and collateral circulation, and altered neurohormonal and autonomic influences. They may contribute to distal embolization during primary PCI, resulting in the no-reflow phenomenon. 

One important limitation of the present retrospective study is the fact that although the level of peak creatine kinase MB isoenzyme and location of IRA occlusion may present an estimation of the infarct area size and the severity of infarction in our study, we might have obtained more accurate data if we had used other cardiac markers’ profiles and echocardiographic or ECG surrogates of infarct sizing. In addition, we failed to include time to PCI from the development of chest pain and time to patency of the artery; nevertheless, all our patients underwent PCI within 12 hours of their symptom initiation.

## Conclusion

According to the results of the present study, older age, higher numbers of Q waves in the surface ECG, not using statin, longer target lesion lengths, higher thrombus grades, and higher IRA-SS were independently associated with the occurrence of the no-reflow phenomenon in our patients, who underwent primary PCI within 24 hours of STEMI. These factors may have a potential to predict the no-reflow phenomenon in such cases. 

## Notes:

***This paper should be cited as: ***Alidoosti M, Lotfi R, Lotfi-Tokaldany M, Nematipour E, Salarifar M, Poorhosseini H, Jalali A. Correlates of the “No-Reflow” or “Slow-Flow” Phenomenon in Patients Undergoing Primary Percutaneous Coronary Intervention. J Teh Univ Heart Ctr 2018;13(3):108-114.
